# Decreased visual acuity is related to thinner cortex in cognitively normal adults: cross-sectional, single-center cohort study

**DOI:** 10.1186/s13195-022-01045-0

**Published:** 2022-07-25

**Authors:** Gyule Han, Ji Sun Kim, Yu Hyun Park, Sung Hoon Kang, Hang-Rai Kim, Song Hwangbo, Tae-Young Chung, Hee Young Shin, Duk L. Na, Sang Won Seo, Dong Hui Lim, Hee Jin Kim

**Affiliations:** 1 Department of Ophthalmology, Samsung Medical Center, Sungkyunkwan University School of Medicine, 81 Irwon-ro, Gangnam-gu, Seoul, 06351 Republic of Korea; 2 Department of Neurology, Samsung Medical Center, Sungkyunkwan University School of Medicine, 81 Irwon-ro, Gangnam-gu, Seoul, 06351 Republic of Korea; 3 Alzheimer’s Disease Convergence Research Center, Samsung Medical Center, Seoul, Republic of Korea; 4 Department of Health Sciences and Technology, SAIHST, Sungkyunkwan University, Seoul, Republic of Korea; 5 Center for Health Promotion, Samsung Medical Center, Sungkyunkwan University School of Medicine, Seoul, Republic of Korea; 6 Department of Clinical Research Design & Evaluation, SAIHST, Sungkyunkwan University, Seoul, Republic of Korea; 7 Department of Digital Health, SAIHST, Sungkyunkwan University, Seoul, Republic of Korea

**Keywords:** Cortical thickness, Visual acuity, Dementia

## Abstract

**Background:**

Decreased visual acuity (VA) is reported to be a risk factor for dementia. However, the association between VA and cortical thickness has not been established. We investigated the association between VA and cortical thickness in cognitively normal adults.

**Method:**

We conducted a cross-sectional, single-center cohort study with cognitively normal adults (aged ≥ 45) who received medical screening examinations at the Health Promotion Center at Samsung Medical Center. Subjects were categorized as bad (VA ≤ 20/40), fair (20/40 < VA ≤ 20/25), and good (VA > 20/25) VA group by using corrected VA in the Snellen system. Using 3D volumetric brain MRI, cortical thickness was calculated using the Euclidean distance between the linked vertices of the inner and outer surfaces. We analyzed the association between VA and cortical thickness after controlling for age, sex, hypertension, diabetes, dyslipidemia, intracranial volume, and education level.

**Results:**

A total of 2756 subjects were analyzed in this study. Compared to the good VA group, the bad VA group showed overall thinner cortex (*p* = 0.015), especially in the parietal (*p* = 0.018) and occipital (*p* = 0.011) lobes. Topographical color maps of vertex-wise analysis also showed that the bad VA group showed a thinner cortex in the parieto-temporo-occipital area. These results were more robust in younger adults (aged 45 to 65) as decreased VA was associated with thinner cortex in more widespread regions in the parieto-temporo-occipital area.

**Conclusion:**

Our results suggest that a thinner cortex in the visual processing area of the brain is related to decreased visual stimuli.

**Supplementary Information:**

The online version contains supplementary material available at 10.1186/s13195-022-01045-0.

## Introduction

Visual disturbance is one of the most common complaints in aging. According to a global survey, an estimated 285 million people have visual problems, and two-thirds of them are aged 50 or older [1]. Several studies have suggested that visual acuity (VA) is associated with cognitive impairment. Previous studies showed that decreased VA is a risk factor for dementia [2–4]. Another study in the USA also demonstrated that VA was related to dementia severity [5].

Ample evidence supports the idea that decreased sensory stimuli lead to the degeneration of relevant areas in the brain. A previous study showed that hearing loss [6–9] or central vision loss due to macular degeneration [10–12] could cause neurodegeneration in the brain. On the other hand, reduced visual input in early life may lead to poor development in the visual processing area of the brain which may result in a thinner cortex [13]. Reversely, an inherently thinner visual cortex or dysmaturation of the visual cortex in the developmental phase may lead to decreased VA [14, 15]. However, the association between VA and cortical thickness among individuals without known ocular disease has not been established yet. Currently, the degree of cortical thickness can be measured on brain magnetic resonance imaging (MRI) [16–18]. As cortical atrophy precedes dementia symptoms and individuals with a thinner cortex are at higher risk for dementia [18–21], cortical thickness is an important biomarker and is used as a proxy for neurodegeneration.

Therefore, we assessed the association between VA and cortical thickness in a large cohort of cognitively normal adults (aged ≥ 45). We hypothesized that reduced VA is associated with a thinner cortex, especially in the primary visual cortex as well as in the areas related to visual processing and visual memory. We also expected that the relationship between decreased VA and cortical thickness would be more prominent in younger adults (aged 45–60), because the effect of decreased VA on thinner cortex may be masked by physiological neurodegeneration related to aging in older adults (aged > 65) [22].

## Methods

### Study design and participants

We retrospectively collected data on 2975 subjects aged 45 years or older who underwent a medical screening examination at the Health Promotion Center of Samsung Medical Center (Seoul, Korea) between September 2008 and December 2014. All of them underwent brain MRI including three-dimensional volume images and visual acuity assessment. We excluded the following participants: (1) subjects with significant cognitive impairment defined by an MMSE score below the 16th percentile of age- and education-matched norms (*n* = 169); (2) subjects with incomplete information on hypertension, diabetes, or dyslipidemia (*n* = 1); and (3) subjects whose cortical thickness measurement failed due to head motion artifact (*n* = 49). We finally included data from 2756 subjects for analysis in this study.

### Standard protocol approvals, registrations, and patient consents

This study was approved by the Institutional Review Board at Samsung Medical Center. All methods were conducted in accordance with the approved guidelines. The need for informed consent was waived by the Institutional Review Board at Samsung Medical Center, as we collected retrospective and de-identified data.

### Health screening examination

Health screening examination was conducted by trained health professionals according to the standard protocols at Samsung Medical Center. This included physical examination, laboratory analysis, brain MRI, and VA measurement. A standardized questionnaire was used to collect information related to demographic characteristics including hypertension, diabetes, dyslipidemia, and total duration of formal education, as previously described [23]. Hypertension was defined as systolic blood pressure ≥ 140 mmHg and/or diastolic blood pressure > 90 mmHg, a self-reported history of hypertension, or current use of antihypertensive medication. Diabetes was defined as a fasting serum glucose level greater than 126 mg/dL, self-reported diabetes history, or use of diabetes medications. Dyslipidemia was defined as a fasting total cholesterol level > 200 mg/dL, low-density lipoprotein level > 100 mg/dL, high-density lipoprotein level < 50 mg/dL, triglyceride level > 200 mg/dL, self-reported history of dyslipidemia, or current use of lipid-lowering medication.

### Acquisition of brain MRI data

All participants underwent a 3D volumetric brain MRI. Previous studies have described the details of MRI protocols and the process of cortical thickness measurements [24]. In brief, an Achieva 3.0T MRI scanner (Philips, Best, The Netherlands) was applied to obtain 3D T1 turbo field echo MRI data with the following imaging parameters: sagittal slice thickness of 1.0 mm with 50% overlap, no gap, repetition time of 9.9 ms, echo time of 4.6 ms, flip angle of 8°, and matrix size of 240 × 240 pixels reconstructed to 480 × 480 over a field view of 240 mm. Radiologists initially inspected all MR images for evidence of brain tumors, lobar infarctions (except lacunar infarctions), and hemorrhages (observed as low intensity on T2-weighted images).

### Cortical thickness measurement

T1-weighted MRIs were automatically analyzed with the standard Montreal Neurologic Institute image processing software (CIVET 2.1.0, http://www.bic.mni.mcgill.ca/ServicesSoftware/CIVET). The technique used in this study was previously described [25]. In summary, native MR images were registered into a standardized stereotaxic space using an affine transformation [26]. Using the artificial neural net classifier, the registered and corrected volumes were classified as white matter (WM), gray matter (GM), cerebrospinal fluid (CSF), and background [27]. Using the Laplacian-based automated segmentation with proximities algorithm, we extracted the surfaces of the inner and outer cortices by deforming the spherical mesh onto the gray-white boundaries of each hemisphere. Cortical thickness was calculated using the Euclidean distance between the linked vertices of the inner and outer surfaces, after applying an inverse transformation matrix to the surface of the cortex and reconstructing it in the native space [28]. We used classified tissue information and a skull mask acquired from the T1-weighted image and computed intracranial volume (ICV) to control for brain size [29]. ICV was defined as the sum of the volume of the GM, WM, and CSF, considering the voxel dimension. GM, WM, CSF, and the background within the mask were transformed back into the individual native space. The thicknesses were registered spatially on an unbiased iterative group template by matching the sulcal folding pattern using surface-based registration involving sphere-to-sphere warping to compare the thickness of the corresponding regions among individuals [30]. Global and regional analyses involving the frontal, temporal, parietal, and occipital lobes were performed with the SUMA program (http://afni.nimh.nih.gov) [7, 31].

### Visual acuity (VA) assessment

VA was measured using the Snellen system. For individuals who wear spectacles, spectacle-corrected VA was recorded. The study subjects were classified into 3 groups based on VA in the better-seeing eye: bad (VA ≤ 20/40), fair (20/40 < VA ≤ 20/25), and good (VA > 20/25) VA. The World Health Organization uses a threshold of 20/60 for visual impairment, while 20/40 is used as the standard of visual impairment for research purposes [32–34]. VA ≤ 20/25 (loss of 1 line) indicates mild poor vision, which means the individual may have mild difficulty in daily life without spectacles [35, 36].

### Statistical analysis

We compared demographic factors across the three groups (bad, fair, and good VA) using chi-square testing or analysis of variance.

To evaluate the association between VA and the cortical thickness of each lobe, we performed a multivariable linear regression analysis. VA group was analyzed as an ordinary variable, and the good VA was used as the reference standard. In addition, multivariable linear regression analysis was conducted using VA as a continuous variable. A subgroup analysis was performed after stratifying subjects into younger adults (aged 45 to 65) and older adults (aged > 65). All analyses were performed with adjustment for age, sex, hypertension, diabetes, dyslipidemia, education, and ICV. We selected these factors as the covariates for the multivariable linear regression based on the clinical relevance.

Analyses were performed using STATA version 15 (StataCorp. 2017. Stata Statistical Software: Release 15. College Station, TX: StataCorp LLC). Reported *p*-values were calculated as two-sided, and the significance level was set at 0.05.

The MATLAB-based toolbox 2014b (https://kr.mathworks.com/products/matlab.html) was used to evaluate the topography of cortical thickness related to VA. A detailed method was previously reported [37]. In brief, after diffusion smoothing to blur each cortical thickness map (leading to increased signal-to-noise ratio and statistical power), linear regression was performed vertex-by-vertex after controlling for age, sex, hypertension, diabetes, dyslipidemia, education, and ICV. Then, we analyzed the localized differences and the statistical map of cortical thickness on the surface model. The thresholds of the statistical maps were set using a false discovery rate (FDR) with a *q*-value of 0.05 after pooling the *p*-values from regression analysis.

## Results

### Demographics of subjects

Among the 2756 subjects, 159 subjects were classified into the bad VA group, 1256 subjects were classified into the fair VA group, and 1341 subjects were classified into the good VA group. Compared to subjects in the bad VA or fair VA groups, subjects in the good VA group were younger, more likely to be male, and had higher education levels and larger ICVs (Table 1). The distribution of VA according to age was plotted in Additional file 1: Fig. S1.

**Table 1:** Baseline characteristics of participants in each visual acuity (VA) group^a^ (total *n* = 2756)

	Bad (*n* = 159)	Fair (*n* = 1256)	Good (*n* = 1341)	*p*^b^			
				Overall	Bad vs fair	Bad vs good	Fair vs good
Age (years)	67.4 ± 7.6	64.9 ± 7.0	61.4 ± 6.6	< 0.001	< 0.001	< 0.001	< 0.001
Sex (male, %)	54 (34.0)	543 (43.2)	786 (58.6)	< 0.001	0.026	< 0.001	< 0.001
Education (years)	10.2 ± 5.1	12.2 ± 4.4	13.6 ± 3.6	< 0.001	< 0.001	< 0.001	< 0.001
Hypertension (%)	82 (51.6)	620 (49.4)	547 (40.8)	< 0.001	0.600	0.009	< 0.001
Diabetes (%)	34 (21.4)	198 (15.8)	206 (15.4)	0.143	0.071	0.050	0.777
Dyslipidemia (%)	41 (25.8)	418 (33.3)	420 (31.3)	0.132	0.057	0.153	0.286
ICV (mL) × 10^5^	13.3 ± 1.5	13.4 ± 1.2	13.8 ± 1.2	< 0.001	0.136	< 0.001	< 0.001
MMSE^c^	26.4 ± 0.3	27.6 ± 0.1	28.2 ± 0.1	< 0.001	< 0.001	< 0.001	< 0.001
VA (logMAR)	0.49 ± 0.13	0.16 ± 0.07	− 0.02 ± 0.05	< 0.001	< 0.001	< 0.001	< 0.001
Total cortical thickness (× 10^−1^mm)	60.16 ± 2.42	60.81 ± 2.29	61.16 ± 2.13	< 0.001	0.001	< 0.001	< 0.001

*ICV* intracranial volume, *MMSE* Mini-Mental state examination, *logMAR* -log (Snellen visual acuity)

^a^Grouped by VA in better-seeing eye: bad = VA ≤ 20/40, fair = 20/40 < VA ≤ 20/25, and good = VA > 20/25 (VA was presented in the Snellen system). Numerical continuous parameters were described as mean ± standard deviation, and categorical parameters were described as total numbers (percentages)

^b^Results from analysis of variance (ANOVA) with Bonferroni post hoc test for continuous variables (total cortical thickness, age, education, ICV, MMSE) and Pearson’s chi-squared test for binary variable (sex, hypertension, diabetes, dyslipidemia)

^c^MMSE has a missing value: 11 subjects in the bad VA group, 54 subjects in the fair VA group, and 58 subjects in the good VA group

### Association between VA and cortical thickness

Compared to the good VA group, subjects in the bad VA group showed reduced cortical thickness with statistical significance (*ß* = − 0.46, 95% confidence interval [CI] − 0.84 to − 0.09) especially in the parietal (*ß* = − 0.57, 95% CI − 1.05 to − 0.10) and occipital (*ß* = − 0.54, 95% CI − 0.96 to − 0.12) lobes in the multivariable linear regression analysis after adjusting for age, sex, hypertension, diabetes, hyperlipidemia, education, and ICV. *p* for trend also showed a statistically significant correlation between VA and global cortical thicknesses, as well as parietal and occipital lobe thickness (*p* = 0.036, 0.023, and 0.025, respectively). However, there was no statistical difference in cortical thickness between the good and fair VA groups (Table 2). The results of the subgroup analysis of corrected VA and uncorrected VA are presented in Additional file 2: Table S1.

**Table 2:** Analysis of the relationship between cortical thickness (x 10^-1^mm) and visual acuity (VA) groups^a^

	Global cortical thickness		Frontal lobe		Temporal lobe		Parietal lobe		Occipital lobe	
	*ß* (95%CI)	*p*^b^	*ß* (95%CI)	*p*^b^	*ß* (95%CI)	*p*^b^	*ß* (95%CI)	*p*^b^	*ß* (95%CI)	*p*^b^
**All age**										
Bad	− 0.46 (− 0.84, − 0.09)	**0.015**	− 0.31 (− 0.70, 0.09)	0.131	− 0.50 (− 1.06, 0.06)	0.079	-0.57 (-1.05, -0.10)	**0.018**	-0.54 (-0.96, -0.12)	**0.011**
Fair	0.01 (− 0.16, 0.19)	0.893	< 0.001 (− 0.19, 0.18)	0.958	− 0.04 (− 0.30, 0.22)	0.780	0.08 (-0.14, 0.30)	0.497	0.02 (-0.17, 0.22)	0.827
Good	Ref.		Ref.		Ref.		Ref.		Ref.	
*p*^c^	**0.036**		0.296		0.210		**0.023**		**0.025**	
*p*^d^			0.296		0.280		**0.050**		**0.050**	
**Age ≤ 65**										
Bad	− 0.71 (− 1.24, − 0.17)	**0.010**	− 0.46 (− 1.03, 0.10)	0.108	− 0.63 (− 1.46, 0.19)	0.130	− 0.83 (− 1.54, − 0.13)	**0.021**	− 0.99 (− 1.60, − 0.39)	**0.001**
Fair	− 0.04 (− 0.25, 0.17)	0.696	− 0.05 (− 0.27, 0.18)	0.689	− 0.20 (− 0.52, 0.13)	0.235	0.12 (− 0.16, 0.40)	0.397	− 0.04 (− 0.28, 0.19)	0.722
Good	Ref.		Ref.		Ref.		Ref.		Ref.	
*p*^c^	**0.035**		0.273		0.209		**0.030**		**0.005**	
*p*^d^			0.273		0.273		0.060		**0.002**	
**Age > 65**										
Bad	− 0.14 (− 0.68, 0.40)	0.615	− 0.09 (− 0.68, 0.50)	0.769	− 0.05 (− 0.82, 0.72)	0.897	− 0.36 (− 1.01, 0.29)	0.278	− 0.08 (− 0.69, 0.53)	0.803
Fair	0.15 (− 0.15, 0.46)	0.327	0.10 (− 0.24, 0.43)	0.572	0.31 (− 0.12, 0.74)	0.158	0.03 (− 0.33, 0.40)	0.858	0.18 (− 0.16, 0.53)	0.299
Good	Ref.		Ref.		Ref.		Ref.		Ref.	
*p*^c^	0.385		0.726		0.278		0.445		0.449	
*p*^d^			0.726		0.599		0.599		0.599	

*CI* confidential interval, presented as ([lower value], [upper value])

^a^Grouped by VA in better-seeing eye: bad = VA ≤ 20/40, fair = 20/40 < VA ≤ 20/25, and good = VA > 20/25 (VA was presented in the Snellen system)

^b^Result from multivariable linear regression adjusted for age, sex, hypertension, diabetes, dyslipidemia, intracranial volume, and education

^c^*p* for trends

^d^Adjusted *p* using Benjamini and Hochberg’s method for the multiple tests

**Fig. 1:**
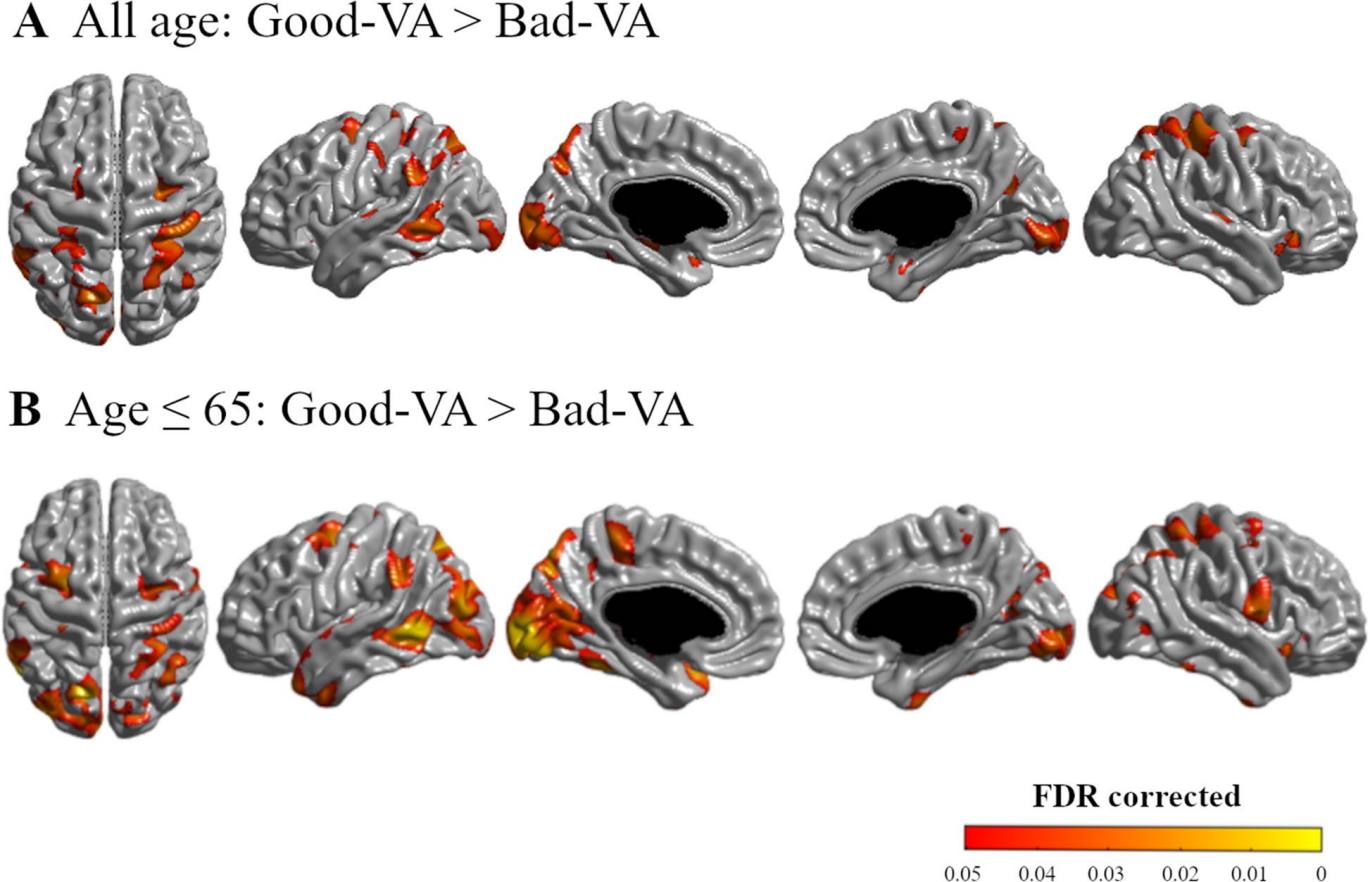
Statistical topography of the relationship between cortical thickness and visual acuity (VA). Compared to the good VA group, the bad VA group showed a thinner cortex in the bilateral temporo-parieto-occipital lobes (**A**). In the analysis of the subjects who were equal or under 65 years old, a similar result was found with a larger extent and greater significance (**B**). False discovery rate corrected (*q* < 0.05). This result was adjusted for age, sex, hypertension, diabetes mellitus, hyperlipidemia, education, and intracranial volume. The MATLAB-based toolbox 2014b (https://kr.mathworks.com/products/matlab.html) was used for the drawing and the configuration of this figure

The statistical topographic map also showed that VA was associated with cortical thickness in the colored areas in Fig. 1. Compared to the good VA group, subjects in the bad VA group had significantly thinner cortices, mainly in the parieto-temporo-occipital areas (bilateral superior parietal lobule, bilateral cuneus, and left lateral temporal areas) and in a focal region in the bilateral medial temporal areas (FDR corrected, *q* < 0.05) (Fig. 1A). However, the uncolored areas showed no significant difference between the good VA group and bad VA group (Fig. 1A).

### Association between VA and cortical thickness in younger and older adults

We also analyzed the association between VA and cortical thickness after stratifying the subjects into younger (45 ≤ age ≤ 65) and older (age > 65) adults. In younger adults, the bad VA group showed a thinner cortex in the global area (*ß* = − 0.71, 95% CI − 1.24 to − 0.17), as well as the parietal (*ß* = − 0.83, 95% CI − 1.54 to − 0.13) and the occipital (*ß* = − 0.99, 95% CI − 1.60 to − 0.39) lobes compared to the good VA group. *p* for trend showed a significant relationship between cortical thickness and VA in the same area (*p* = 0.035, .030, and 0.005, respectively). However, in older adults, the cortical thickness of the bad VA group and fair VA group did not differ significantly from that of the good VA group (Table 2).

On the statistical map of younger adults, the bad VA group showed a thinner cortex compared to the good VA group mainly in the parieto-temporo-occipital areas, including the bilateral lateral parietal area; left precuneus, bilateral cuneus, and left lateral temporal areas; and also a focal region in the bilateral medial temporal areas (FDR corrected, *q* < 0.05) (Fig. 1B). However, the uncolored areas showed no significant difference between the good VA group and bad VA group in younger adults (Fig. 1B). In older adults, however, the statistical map of cortical thickness in the bad VA group and fair VA group did not differ from that of the good VA group.

## Discussion

We evaluated the association between VA and cortical thickness in a large cohort of cognitively normal adults. We found that decreased VA was related to a thinner cortex mostly in the posterior regions—specifically, the parieto-temporo-occipital areas. This relationship was more robust in younger adults but was not seen in older adults. Our results imply that structural alterations in the visual processing area of the brain are related to decreased VA in cognitively normal healthy adults.

The major finding in this study was that decreased VA was associated with thinner cortex in cognitively normal adults. Although the criteria for the bad VA group in this study did not reach the visual impairment standard defined by the WHO (visual acuity ≤ 20/60), decreased VA was significantly correlated with a thinner cortex. The topography showed that this correlation was most significant in the posterior regions, specifically in the parieto-temporo-occipital area. This area is known to be responsible for processing visual information. Visual information primarily reaches the occipital lobe and then follows two streams to process this information: the dorsal stream (known as the “where pathway”) and the ventral stream (known as the “what pathway”). The “what pathway,” which spreads from the primary visual cortex to the temporal lobe, performs serial processing of perception and recognition of shape, size, objects, faces, and text [38]. On the other hand, the “where pathway,” which spreads from the primary visual cortex to the parietal lobe, processes spatial layout such as location, distance, relative position, position in egocentric space, and motion [38]. Our results suggest that the degree of visual stimuli might affect the cortical thickness in corresponding areas. We also observed that the bad VA group had a thinner cortex in the bilateral medial temporal areas compared to the good VA group. We expected the right medial temporal lobe, which is responsible for visual memory, to be more associated with decreased VA as previously suggested [39]. However, our results showed that both medial temporal lobes were associated with decreased VA. Such results suggested that brain structural alteration related to decreased VA is not limited to visual processing areas, but extends to regions related to visual and verbal memory. A previous Japanese study also showed a possible link between VA and cognitive impairment, even in cognitive domains unrelated to visuospatial function or visual memory [40].

A thinner cortex related to decreased VA was more robust in younger adults in this study. Because aging is the major factor that affects brain cortical atrophy [41], we stratified by age to minimize the effect of aging on cortical atrophy. Consequently, the relationship between VA and cortical thickness was more significant in younger adults. In older adults, there was no significant association. It is possible that reduced VA itself may not be a significant factor of cortical atrophy in older adults. It is also possible that, in older adults, other factors might have comprehensively affected cortical atrophy and may have masked the effect of decreased VA although we adjusted for possible confounders [42–49]. Another possible explanation would be neuroplasticity. That is, better VA might induce a thicker cortex especially in younger adults who are less affected by aging.

This study is the first report that included a topographical analysis of the relationship between a thinner cortex and decreased VA in cognitively normal adults. Similar studies have been conducted on patients with visual problems with known ocular diseases such as glaucoma, retinal disease, or macular disease [10, 11, 50–56]. These studies suggested that decreased VA from ocular diseases also could cause structural alteration in the brain, and this was pathologically confirmed in a glaucoma patient [57]. Subjects with acquired blindness were reported to have a thinner primary visual cortex [10, 11, 50–55, 58]. Our study showed that cortical atrophy patterns related to decreased VA were similar to those from studies on acquired blindness. These studies imply that decreased visual stimuli from various conditions can be related to structural changes in the brain. Considering that most of the participants in this study had no specific ocular diseases such as glaucoma or macular disease, our study inferred that a thinner cortex was related to decreased VA per se.

The pathophysiologic mechanism of the relationship between a thinner cortex and decreased VA remains unclear. One possible mechanism is desensitization. Decreased VA, which decreases visual stimuli to the brain, can cause anterograde trans-synaptic degeneration. This has been reported in visual cortices of patients with retinal disease/damage [59]. Retrograde trans-synaptic degeneration has also been observed: when the brain cortex was damaged, the number of retinal ganglion cells was reduced in regions that projected nerve fibers to the damaged cortex [51–53, 60, 61]. Furthermore, decreased visual stimuli also can reduce functional connectivity between the visual cortex and somatosensory area and between the visual cortex and temporal cortex, which was seen in a study of subjects with recently acquired blindness [62].

Due to the retrospective and cross-sectional nature of the study, our data does not provide a causal relationship between decreased VA and thinner cortex. Decreased VA might have affected neurodegeneration in the brain, or decreased VA might have affected poor development in the visual processing area of the brain, or reversely, an inherently thinner visual cortex or dysmaturation of the visual cortex in the developmental phase might have caused decreased VA.

### Limitations

Our study has several limitations. Firstly, due to the retrospective and cross-sectional nature of our study, we could not elucidate whether the association between visual impairment and thinner cortex had a causal relationship. The lack of serum or CSF biomarkers of neurodegeneration limits the interpretation of our results as to whether a thinner cortex was due to neurodegenerative change. A further longitudinal prospective study is needed to see whether decreased VA at baseline causes a thinner cortex over time or whether longitudinal VA change is related to a thinner cortex. Secondly, we could not completely exclude individuals who had ocular diseases such as glaucoma or macular degeneration. Although we acknowledge the association between neurodegeneration and ocular diseases, the prevalence of these diseases is relatively small in the general population [63, 64]. Thus, we suspect that the significant result of a thinner cortex in this study was mostly derived from the effect of low VA itself. Third, the number of the participants in bad VA group was much smaller than the fair or good VA group. This imbalance might have led to artifactual differences related to unknown confounders. Lastly, a multi-center study in various regions or ethnicity is required to see consistent results for generalization.

## Conclusion

Our results suggest that a thinner cortex in the visual processing area of the brain is related to decreased visual stimuli in cognitively normal adults.

## Supplementary Information

**Additional file 1: Fig. 1.** The distribution of visual acuity according to the age of the study participants. Grouped by VA in better-seeing eye: bad = VA ≤ 20/40, fair = 20/40 < VA ≤ 20/25, good = VA > 20/25 (VA was presented in Snellen system).

**Additional file 2: Table S1.** Subgroup analysis of the relationship between cortical thickness (x 10-1mm) and visual acuity (VA) groups divided by VA types.

## Data Availability

The datasets used and/or analyzed during the current study are available from the corresponding authors (DHL, HJK) on reasonable request.

## Abbreviations

VA: Visual acuity
WM: White matter
GM: Gray matter
CSF: Cerebrospinal fluid
ICV: Intracranial volume
logMAR: Logarithm of the minimum angle of resolution
MMSE: Mini-Mental State Examination
