# Supplementary figures and images for: Decreased visual acuity is related to thinner cortex in cognitively normal adults: cross-sectional, single-center cohort study
Source: Alzheimers Res Ther. 2022 Jul 25;14:99. doi: 10.1186/s13195-022-01045-0 (PMC9310451; doi:10.1186/s13195-022-01045-0)

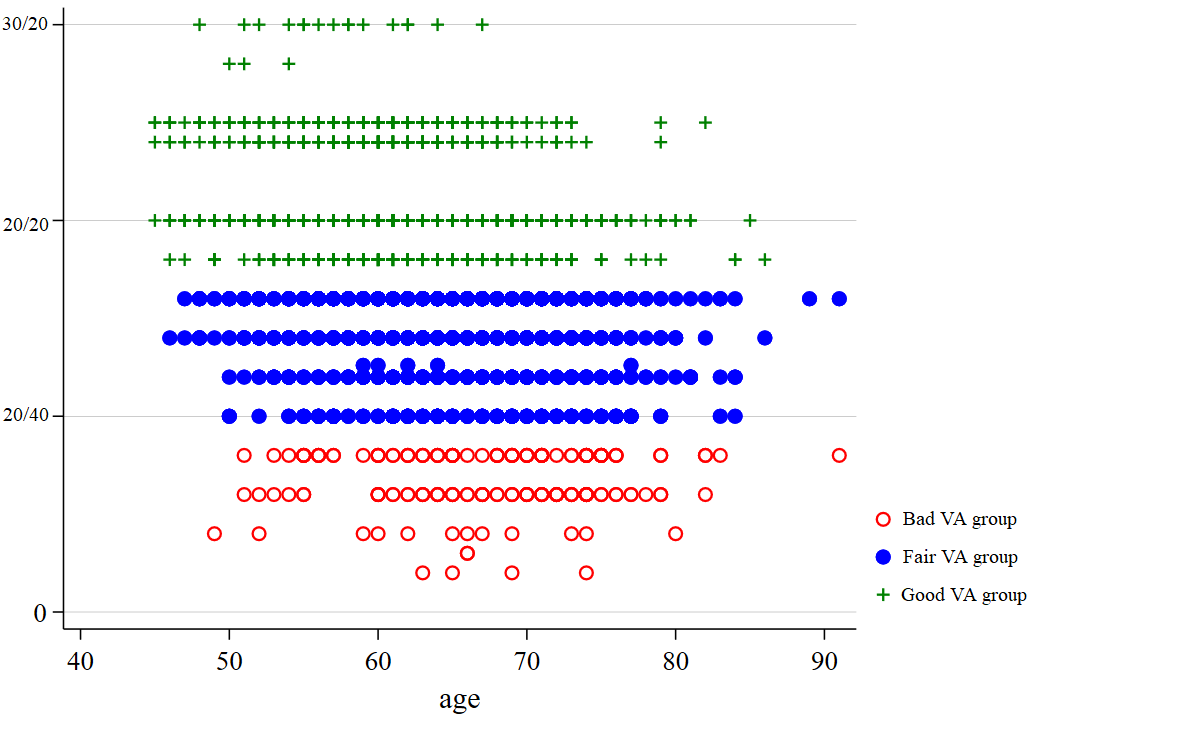

Supplement: Supplementary file 1 — Additional file 1: Fig. 1. The distribution of visual acuity according to the age of the study participants. Grouped by VA in better-seeing eye: bad = VA ≤ 20/40, fair = 20/40 < VA ≤ 20/25, good = VA > 20/25 (VA was presented in Snellen system). [file 13195_2022_1045_MOESM1_ESM.tif]
