# Supplementary material for: Decreased visual acuity is related to thinner cortex in cognitively normal adults: cross-sectional, single-center cohort study
Source: Alzheimers Res Ther. 2022 Jul 25;14:99. doi: 10.1186/s13195-022-01045-0 (PMC9310451; doi:10.1186/s13195-022-01045-0)
Supplement: Supplementary file 2 — Additional file 2: Table S1. Subgroup analysis of the relationship between cortical thickness (x 10-1mm) and visual acuity (VA) groups divided by VA types. [file 13195_2022_1045_MOESM2_ESM.docx]

|  | **Global  cortical thickness** | | **Frontal lobe** | | **Temporal lobe** | | **Parietal lobe** | | **Occipital lobe** | |
| --- | --- | --- | --- | --- | --- | --- | --- | --- | --- | --- |
|  | ß (95%CI) | p^b^ | ß (95%CI) | p^b^ | ß (95%CI) | p^b^ | ß (95%CI) | p^b^ | ß (95%CI) | p^b^ |
| **Uncorrected VA (N = 1,985)** | |  |  |  |  |  |  |  |  |  |
| Bad | -0.46 (-0.85, -0.07) | **0.022** | -0.35 (-0.76, 0.07) | 0.106 | -0.41 (-1.00, 0.17) | 0.166 | -0.54 (-0.10, -0.04) | **0.032** | -0.50 (-0.94, -0.06) | **0.027** |
| Fair | 0.01 (-0.20, 0.21) | 0.949 | -0.03 (-0.25, 0.19) | 0.786 | 0.04 (-0.26, 0.34) | 0.788 | 0.05 (-0.22, 0.31) | 0.733 | 0.03 (-0.20, 0.26) | 0.779 |
| Good | Ref. | | Ref. | | Ref. | | Ref. | | Ref. | |
| p^c^ | **0.050** | | 0.260 | | 0.283 | | 0.054 | | **0.047** | |
| p^d^ |  | | 0.078 | | 0.078 | | 0.078 | | 0.078 | |
| **Corrected VA (N = 771)** | |  |  |  |  |  |  |  |  |  |
| Bad | -0.54 (-1.75, 0.66) | 0.376 | -0.62 (-1.90, 0.66) | 0.342 | -1.04 (-0.29, 0.81) | 0.270 | 0.02 (-1.58, 1.62) | 0.982 | -0.38 (-1.80, 1.04) | 0.598 |
| Fair | -0.16 (-0.49, 0.16) | 0.310 | -0.13 (-0.47, 0.22) | 0.473 | -0.35 (-0.84, 0.14) | 0.170 | -0.03 (-0.45, 0.40) | 0.897 | -0.19 (-0.57, 0.20) | 0.329 |
| Good | Ref. | | Ref. | | Ref. | | Ref. | | Ref. | |
| p^c^ | 0.376 | | 0.342 | | 0.270 | | 0.982 | | 0.598 | |
| p^d^ |  | | 0.078 | | 0.078 | | 0.078 | | 0.078 | |

**Supplementary Table 1.** Subgroup analysis of the relationship between cortical thickness (x 10^-1^mm) and visual acuity (VA) groups^a^ divided by VA types.

*CI* confidential interval, presented as ([lower value], [upper value]).

^a^ Grouped by VA in better-seeing eye: bad = VA ≤ 20/40, fair = 20/40 < VA ≤ 20/25, good = VA > 20/25 (VA was presented in Snellen system)

^b^ Result from multivariable linear regression adjusted for age, sex, hypertension, diabetes, dyslipidemia, intracranial volume, and education.

^c^ P for trends.

^d^Adjusted P using Benjamini and Hochberg’s method for the multiple tests
